# A novel calreticulin of *Psoroptes ovis* regulated keratinocyte function resulting in host skin barrier dysfunction: implications for involvement in the pathogenesis of psoroptic mange

**DOI:** 10.1186/s13071-025-06800-4

**Published:** 2025-05-30

**Authors:** Yane Li, Guiying Hao, Je Fan, Fangyan Wu, Xiangyue Yao, Youping Liang, Jing Xu, Ran He, Hui Wang, Yue Xie, Xiaobin Gu

**Affiliations:** 1 Department of Parasitology, College of Veterinary Medicine, Sichuan Agricultural University, Chengdu, Sichuan People’s Republic of China; 2 School of Animal Science, Xichang College, Xichang, People’s Republic of China

**Keywords:** *Psoroptes ovis*, Calreticulin, Tissue localization, Keratinocytes, Immune regulation

## Abstract

**Background:**

*Psoroptes ovis*, the causative agent of psoroptic mange, affects a wide range of domestic and wild animals, causing substantial economic losses and threatening wildlife survival. However, the underlying pathogenesis of this ectoparasitic disease remains poorly understood.

**Methods:**

In this study, we comprehensively characterized the sequence conservation and excretory–secretory properties of *P. ovis* calreticulin (PsoCRT) using sequence alignment, immunoblotting, and immunofluorescence assays. To investigate the functional impact of recombinant PsoCRT (rPsoCRT), we conducted in vitro studies assessing its effects on keratinocyte proliferation, migration, differentiation, and the expression of immune regulatory factors. In addition, we employed rabbit ear intradermal injections of rPsoCRT to histologically observe tissue changes and confirm alterations in the expression profiles of immune regulatory factors.

**Results:**

PsoCRT was expressed across all developmental stages of *P. ovis*, with peak expression observed in adult males. Notably, PsoCRT was excreted and secreted into the host epidermis, primarily localizing within the stratum granulosum and spinosum. Intriguingly, sera from rabbits infested with *P. ovis* did not recognize PsoCRT. In vitro studies revealed that rPsoCRT significantly inhibited keratinocyte proliferation and migration, promoted differentiation, and upregulated the expression of interleukin (IL)-1β, IL-6, IL-36, C–C motif chemokine ligand 27 (CCL27), and vascular endothelial growth factor (VEGF) in vitro, without altering the levels of interferon (IFN)-γ or tumor necrosis factor (TNF)-α. In vivo, rabbit ear intradermal injections of rPsoCRT induced epidermal cell differentiation, immune cell infiltration, and an upregulation of IL-6, CCL27, and VEGF expression.

**Conclusions:**

PsoCRT disrupted the physical and immune barriers of keratinocytes, leading to skin dysfunction and facilitating a microenvironment conducive to *P. ovis* parasitization, thereby highlighting its important role in the pathogenesis of psoroptic mange.

**Graphical abstract:**

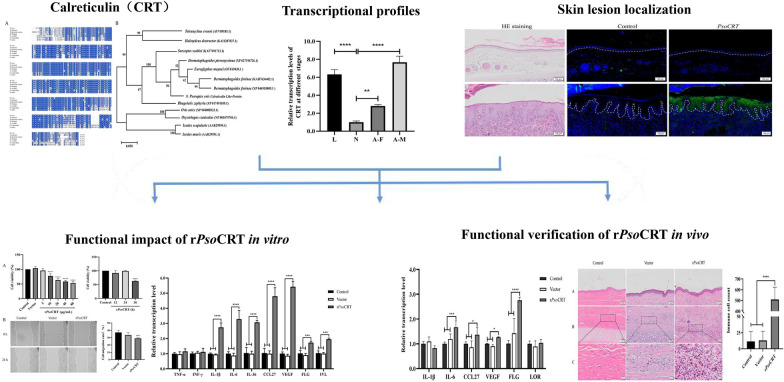

## Background

*Psoroptes ovis*, the causative agent of psoroptic mange, is a prevalent ectoparasite affecting both domestic and wild animals worldwide. Its clinical manifestations, including emaciation, scabbing, and skin thickening, ultimately result in significant economic losses and severe welfare concerns in the livestock industry [1–3]. Despite this substantial impact on both economic and animal welfare, our understanding of the pathogenesis of psoroptic mange remains limited. As a non-burrowing mite, *P. ovis* resides on the host’s skin surface [4], utilizing its mouthparts to abrade the epidermal layer without penetrating it [5, 6]. Consequently, keratinocytes, the predominant cell type in the epidermis, become the primary targets of interaction between the mite and its host [5, 7]. Keratinocytes, as the fundamental units of the skin’s epidermal barrier, play a crucial role in maintaining the structural and functional integrity of the skin. Through their unique ability to synthesize and secrete a variety of bioactive molecules, they finely regulate the skin’s immune response, hydration levels, and permeability, thereby reinforcing the skin’s protective barrier against external threats [8, 9]. Importantly, keratinocytes, as the front line cells directly interacting with *P. ovis*, are exposed to numerous antigenic molecules secreted and excreted by the mite during its parasitic infestation [6, 10, 11]. These antigenic molecules are known to disrupt keratinocyte function [7]; however, a substantial portion of the specific protein components that mediate the pathogenic processes of *P. ovis* remain unidentified and uncharacterized to date. This gap in our knowledge hinders the understanding of the host–pathogen interplay and the development of effective therapeutic strategies.

Calreticulin (CRT) is a highly conserved endoplasmic reticulum Ca^2+^-binding protein and lectin-like chaperone [12], ubiquitously across diverse parasites, including protozoa, ectoparasites, and helminths [13]. In parasites, CRT establishes complex interactions with various host target cells, thereby delicately orchestrating the progression of parasite diseases [14]. For instance, *Trichinella spiralis* and *Schistosoma japonicum* have been shown to secrete or express CRT on their surfaces, which modulates cellular immunity, triggers immune shifts, facilitates immune evasion, and participates in other critical biological processes [15, 16]. Through these intricate mechanisms, CRT precisely regulates the initiation of host immune response [13, 17], ultimately aiding in the establishment and maintenance of parasitism within the host.

In a recent study, we identified a novel CRT protein, termed PsoCRT (Genbank accession number: PQ498351), within the excretory–secretory proteins of *P. ovis* [18]. However, the functional characterization of PsoCRT remains elusive. To address this knowledge gap, we conducted a comprehensive analysis of PsoCRT gene transcription expression and its tissue localization in rabbit skin lesions. Furthermore, we evaluated the regulatory effects of rPsoCRT on keratinocytes both in vitro and in vivo using rabbit models. Finally, we discussed the potential role of PsoCRT in the pathogenesis of psoroptic mange.

## Methods

### Animal, cell, and parasite sources

Healthy New Zealand rabbits aged 3 months (*n* = 9) were purchased from a rabbit farm in Sichuan province that had no history of *P. ovis* infestation. In addition, two rabbits infested with *P. ovis* (*n* = 2) were provided by the Department of Parasitology, Sichuan Agricultural University (Sichuan, China). Two healthy female rats aged 6 weeks were obtained from Chengdu Dashuo Laboratory Animal Co., Ltd. Keratinocytes were procured from Zhejiang Meisen Cellular Technology Co. (CTCC) and were cultured in Dulbecco’s modified Eagle medium (DMEM) complete medium, supplemented with 10% (*v*/*v*) fetal bovine serum (FBS) and 1% (*v*/*v*) penicillin–streptomycin. The cells were then incubated at 37 ℃ for 24 h in a humidified atmosphere with 5% CO_2_.

*Psoroptes* mites were collected from infested rabbits to maintain mite colonies at the Department of Parasitology, Sichuan Agricultural University. Mites at each lifecycle stage (larva, nymph, male and female) were individually harvested, and a mixed population comprising mites from all lifecycle stages was also collected, following a previously established method [19].

### Prediction of CD4^+^ T-cell epitopes, homology analysis, and construction of an evolutionary tree

CD4^+^ T-cell epitopes were predicted by the Immune Epitope Database (IEDB) Analysis Resource (http://tools.immuneepitope.org/CD4episcore/). Multiple sequence alignment of PsoCRT and its orthologs was performed using DNAMAN version 9.0 (Lynnon Biosoft, Quebec, Canada). A phylogenetic tree was inferred using the neighbor-joining (NJ) method, based on the Poisson correction method, with MEGA version 7.0 (bootstrap = 1000).

### Preparation of rPsoCRT, excretory–secretory (E/S) proteins, and whole-body proteins from *P. ovis* mites

The BL21 (DE3) strain of *Escherichia coli* harboring the pET-32a-PsoCRT plasmid, preserved in the Department of Parasitology, Sichuan Agricultural University, was resuscitated and cultured in Luria–Bertani (LB) medium at 37 ℃ for expansion. Induction was carried out with 1 mmol/L isopropyl-β-D-thiogalactoside (IPTG) for 24 h, followed by cell harvesting via centrifugation. The cells were subsequently lysed using ultrasonication to obtain the supernatant, which underwent purification through Ni^2+^-affinity chromatography to yield the purified rPsoCRT. The rPsoCRT was further treated using an Endotoxin Removal Kit (Smart-Life Sciences Biotechnology Co., Ltd., Changzhou, China) to eliminate endotoxin contamination.

Mixed-stage mites of *P. ovis* were washed three times with sterile water, subsequently sterilized in 70% ethanol, and then centrifuged at 500 × *g* for 30 s to obtain clean mite bodies. Whole-body proteins were extracted from 20 mg of these clean mites using the ExKine™ Pro Animal Cell/Tissue Total Protein Extraction Kit (Abbkine, Wuhan, China). E/S proteins of *P. ovis* were extracted following the methodology described by Warkin CA et al. [7]. Briefly, after obtaining the clean mites through the aforementioned washing process, they were placed in Petri dishes and incubated overnight at 28 °C with 75% relative humidity (± 15%) to facilitate their recovery and the secretion of E/S products. Following the removal of excess mites, eggs, and debris, the mite secretions were collected from the culture dishes using pre-cooled phosphate-buffered saline (PBS) solution. Subsequently, the secretions were precipitated and concentrated using the trichloroacetic acid (TCA)-acetone method to obtain the E/S proteins.

### Polyclonal antibody production and western blotting analysis

To prepare polyclonal antibody against rPsoCRT, rats (*n* = 2) were immunized following the method described by Manunathachar [20]. Briefly, each rat was initially subcutaneously injected with 0.3 mg purified rPsoCRT mixed an equal volume of Freund’s complete adjuvant (Sigma, St. Louis, USA). For the second and third injections at 7 day intervals, Freund’s incomplete adjuvant (Sigma) was used instead. Sera samples were collected before immunization and 3 days after the third immunization. The anti-preimmune immunoglobulin G (IgG) and anti-rPsoCRT IgG were purified from the collected sera using Protein G affinity chromatography (GenScript, Nanjing, China), following the manufacturer’s instructions.

Whole-body extracts, E/S proteins of *P. ovis*, and the purified rPsoCRT were separated by 10% sodium dodecyl sulfate–polyacrylamide gel electrophoresis (SDS-PAGE). The protein bands were then transferred onto a PVDF membrane using Trans-Blot SD Semi-Dry Transer Cell (Bio-rad, California, USA). The membrane was blocked with a 5% (*v*/*v*) solution of skimmed milk in PBS and subsequently incubated overnight at 4 ℃ with either rabbit *P. ovis*-positive or -negative sera, or rat anti-preimmune IgG, or rat anti-rPsoCRT IgG (all diluted 1:200 in PBS). Following this, the membranes were incubated with horseradish peroxidase (HRP)-conjugated goat anti-rabbit/rat antibody (diluted 1:2000) (Absin, Shanghai, China). After washing five times with Tween 20 in Tris-buffered saline (TBST), the membranes were visualized using the Enhanced HRP-DAB Chromogenic Substrate Kit (Tiangen, Beijing, China).

### Transcriptional analysis of calreticulin at different life stages of *P. ovis*

For transcriptional profiling of calreticulin across various life stages of *P. ovis*, total RNA was extracted from mites at each life-cycle stage using the Trizol UP kit (TransGen Biotech, Beijing, China). Subsequently, the RNA was reverse transcribed into cDNA with the RT Easy™ II kit with gDNase Eraser (Foregene, Chengdu, China). Real-time fluorescence quantitative polymerase chain reaction (qRT-PCR) was performed in a 20 μL reaction mixture containing 10 μL of 2× Real PCR Easy™ Mix-SYBR (Foregene), each reaction contained 0.8 μL of each primer (10 μM), 1.5 μL of total cDNA, and 6.9 μL of RNase-free ddH_2_O. The β-actin gene was employed as an internal reference for normalization of calreticulin gene expression levels, with the nymphal stage serving as the baseline control. Primer sequences are listed in Table 1. Each sample was analyzed in triplicate to ensure reproducibility. Relative gene expression levels were calculated using the 2^−ΔΔCt^ method [21].

**Table 1:** Primer sequences for qRT-PCR

Species	Gene	Primer sequence (5′–3′)	Size (bp)
*P.ovis*	β-actin	F: TGAATTGCCTGATGGTCAAG R: TGGCGAACAAGTCTTTACGG	92
	*Pso*CRT PQ498351	F: GCCGATGCAGATTGGTCA R: TGCGATGTTTGTATGCCTTT	120
Human (HaCaT)	GAPDH NM_001256799.3	F: CTTTGTCAAGCTCATTTCCTGG R: TCTTCCTCTTGTGCTCTTGC	133
	IL-1β NM_000576.3	F: AACCTCTTCGAGGCACAAGG R: GTCCTGGAAGGAGCACTTCAT	197
	IL-6 NM_001318095.2	F: TCCTTCTCCACAAACATGTAACAA R: TCACCAGGCAAGTCTCCTCA	143
	TNF-α NM_000594.4	F: GCCCATGTTGTAGCAAACCC R: TGAGGTACAGGCCCTCTGAT	133
	IFN-γ NM_000619.3	F: GAGTGTGGAGACCATCAAGGA R: TGGACATTCAAGTCAGTTACCGAA	114
	IL-36 XM_054341389.1	F: CACCTTCGAGTCTGTGGCTT R: CCACATCTTCCTCCAAAGCTTAAA	149
	VEGF NM_001025367.3	F: CGCAAGAAATCCCGTCCCT R: GCAACGCGAGTCTGTGTTTT	108
	CCL27 NM_006664.4	F: CAGCTCTACCGAAAGCCACT R: GAGCCAGGTGAAGCACGAAA	112
	IVL NM_005547.4	F: GCCTTACTGTGAGTCTGGTTGA R: GACAGGCACCTTCTGGCAT	183
	FLG NM_002016.2	F: CGGCAAATCCTGAAGAATCCA R: GTGCTTTCTGTGCTTGTGTCC	189
Rabbit	β-actin NM001101683. 1	F: GGCATGGAGTCGTGTGGCATC R: CGTGTTGGCGTACAGGTCCTTG	90
	FLG XM_008264430.3	F: TCGTGCAATCCTAAAGAACCC R: ATGCCTTTTGTGCTTTCGCT	190
	IL-1β NM_001082201.1	F: GGCACAACAGATCGCTTTGG R: CAGGTCATTTTCATTGCCACTGT	131
	IL-6 NM_001082064.2	F: GACGACCACGATCCACTTCA R: AGAGCCCATGAAATTCCGCA	115
	CCL27 XM_002707938.4	F: ACAGCCACTGTCAAACAAGC R: GAGCCAGATGAAGCACGAAAG	101
	VEGF XM_051854688.1	F: CGCAAGAAATCCCGTCCCT R: GCCTCGGCTTGTCACATCT	163
	FLG XM_008264430.3	F: TCGTGCAATCCTAAAGAACCC R:ATGCCTTTTGTGCTTTCGCT	190
	LOR XM_002715344.5	F:AAGTCGGCCCGGATCAATAG R: AGGAAGGGGACCCCGAAG	126

### Immunolocalization of CRT in lesional skin of rabbit infested with *P. ovis*

Skin samples were collected via punch biopsy from the lesional ear skin of rabbits naturally infested with *P. ovis* (*n* = 2) and from corresponding healthy skin locations on the rabbits (*n* = 2). These samples were processed into paraffin sections for subsequent hematoxylin and eosin (HE) staining and immunofluorescence analysis. For immunofluorescence staining, the sections were dewaxed, rehydrated, and blocked, followed by overnight incubation at 4 °C with either rat anti-preimmune IgG or anti-rPsoCRT IgG (diluted 1:100 in PBS). After thorough washing, the sections were incubated with fluorescein isothiocyanate (FITC)-labeled goat anti-rat IgG (diluted 1:200 in PBS; Abclonal, Wuhan, China), counterstained with 4′, 6-diamidino-2-phenylindole (DAPI), and visualized using an immunofluorescence microscope (BX53, Olympus, Japan).

### CCK-8 assay

Cell viability was assessed by a Cell Counting Kit-8 assay (Oriscience, Chengdu, China) according to the manufacturer’s instructions. Human keratinocyte (HaCaT) cells (1 × 10^6^ cells/well) were seeded into 96-well plates with different concentrations of rPsoCRT (5, 10, 20, 40, and 80 μg/mL) and incubated for different times (12, 24, and 36 h). Simultaneously, PBS (0.01 mol/L, pH 7.4) and pET-32a protein were set as control groups, respectively. Each treatment was conducted in triplicate. Following the incubation period, 10 μL of CCK-8 solution was added to each well, and the plates were incubated for an additional 2 h. The absorbance at 450 nm was measured using a Benchmark plus microplate reader (Bio-Rad, Hercules, CA). The percentage of viable cells was calculated using the following formula: cell survival rate (%) = [(mean OD_450_ value in test wells − mean OD_450_ value in blank wells)/(mean OD_450_ value in control wells − mean OD_450_ value in blank wells)] × 100.

### Scratch assay

To assess cell migration in HaCaT cells, 1 × 10^6^ cells/well were plated in a 12-well plate and incubated until reaching 80% confluence at 37 ℃ in a 5% CO_2_ incubator (Thermo, Waltham, USA). Following three washes with PBS, a uniform scratch was created across the cell monolayer using a sterilized 200 μL pipette tip. Detached cells were carefully removed, and the remaining cells were co-incubated with the optimal concentration of rPsoCRT determined in the CCK-8 assay for an additional 24 h. Simultaneously, two control groups were set up: one with PBS (0.01 mol/L, pH 7.4) and the other with pET-32a protein at the same concentration as rPsoCRT. Each treatment was performed in triplicate. Cell migration was evaluated by capturing images of three randomly selected, non-overlapping fields using an inverted microscope (ICX41, Ningbo Sunny Instruments Co., Ltd., China) and quantifying the migration area using ImageJ 1.53e software. The cell migration rate (%) was calculated as follows: (initial scratched area − scratched area after incubation)/initial scratched area × 100.

### qRT-PCR analysis of immunoregulatory factors and differentiation proteins in HaCaT cells following rPsoCRT treatment

HaCaT cells (1 × 10^6^ cells/well) were plated in 12-well plates and treated with rPsoCRT (5 μg/ml) or controls (PBS, pET-32a at 5 μg/ml) once they reached 70% confluence. The cells were then incubated for 24 h at 37 °C in a 5% CO_2_ atmosphere. All treatments were performed in replicates. Total RNA was extracted from HaCaT cells using a Tissue/Cell RNA Extraction Kit (Foregene) and subsequently reverse transcribed into cDNA using a Reverse Transcription Kit (Foregene). qRT-PCR reactions were performed using Real Time PCR Easy™-SYBR Green I (Foregene) to quantify the relative transcription levels of involucrin (IVL), filaggrin (FLG), tumor necrosis factor-α (TNF-α), interferon-γ (IFN-γ), interleukin-1 beta (IL-1β), interleukin-6 (IL-6), interleukin-36 (IL-36), C–C motif chemokine ligand 27 (CCL27), and vascular endothelial growth factor (VEGF). Glyceraldehyde-3-phosphate dehydrogenase (GAPDH) was used as the internal reference gene, with the PBS-treated group serving as the control for normalization. Each qRT-PCR reaction mixture contained 0.8 μL of each primer, 10 μL of 2× Real PCR Easy™ Mix-SYBR (Foregene), 1.5 μL of cDNA, and ddH_2_O up to a total volume of 20 μL. The relative expression levels of the targets genes were calculated using the 2^−ΔΔCt^ method [21]. The primer sequences are listed in Table 1.

### Histopathological analysis of rabbit ear skin following rPsoCRT injection using HE staining

Nine 3-month-old healthy New Zealand White rabbits were used in this study, with three animals assigned to each experimental group. One day prior to the experiment, the dorsal surface of their ears were shaved and cleaned. A single injection site on the dorsal surface of each ear was selected for intradermal injections of 100 μg of rPsoCRT, 100 μL of PBS (as a negative control), or 100 μg of pET-32a empty vector (as an additional control). Twenty-four hours post-injection, the rabbits were euthanized, and skin tissues encompassing the injection sites were carefully excised and fixed in 4% paraformaldehyde for subsequent paraffin embedding. The embedded tissues were then sectioned and stained with hematoxylin and eosin (HE). The stained sections were scanned using an Olympus VS120 S6 pathology scanner (OLYMPUS, Tokyo, Japan). Six random fields of view from each injection site were examined under a microscope, and immunocompetent cells were quantified.

### qRT-PCR detection of immunoregulatory factors and differentiation proteins in epidermis of rabbit skin tissues following rPsoCRT injection

Epidermal tissues were isolated from rabbit skin collected at the aforementioned residual injection sites. The tissues were carefully dissected to remove excess subcutaneous fat and muscular tissues, followed by thorough rinsing with D-Hanks buffer. Subsequently, the skin samples were subjected to overnight digestion at 4 °C in 0.4% Dispase II solution, with the epidermis facing downwards. The following day, the epidermal tissue layer was gently separated in a sterile culture dish.

Total RNA was extracted from the isolated epidermal tissue using the Tissue/Cell RNA Extraction Kit (Foregene), and reverse transcribed into cDNA with the Reverse Transcription Kit (Foregene), adhering to the protocols previously described in this study. qRT-PCR analysis was then conducted using Real Time PCR EasyTM-SYBR Green I (Foregene), with β-actin serving as the internal control and the PBS injected group as the reference. The mRNA transcription levels of FLG, LOR, IL-1β, IL-6, CCL27, and VEGF were quantified. The reaction mixture consisted of 0.8 μL of each primer, 10 μL of SYBR qRT-PCR™ Master Mix, 1.5 μL of cDNA template, and ddH_2_O to a final volume of 20 μL. Relative gene expression was calculated using the 2^−ΔΔCt^ method [21]. The primer sequences are detailed in Table 1.

### Statistical analysis

Statistical differences between groups were evaluated using one-way analysis of variance (ANOVA). Data are presented as the means ± standard deviation (SD). Significance level was set at *P < 0.05, **P < 0.01, ***P < 0.001, and ****P < 0.0001. All experiments were conducted with a minimum of three biological replicates. Data processing and graphical representation were performed using GraphPad Prism 8.0 software.

## Results

### Prediction of PsoCRT CD4^+^ T-cell epitopes, homology analysis, and evolutionary tree construction

**Fig. 1:**
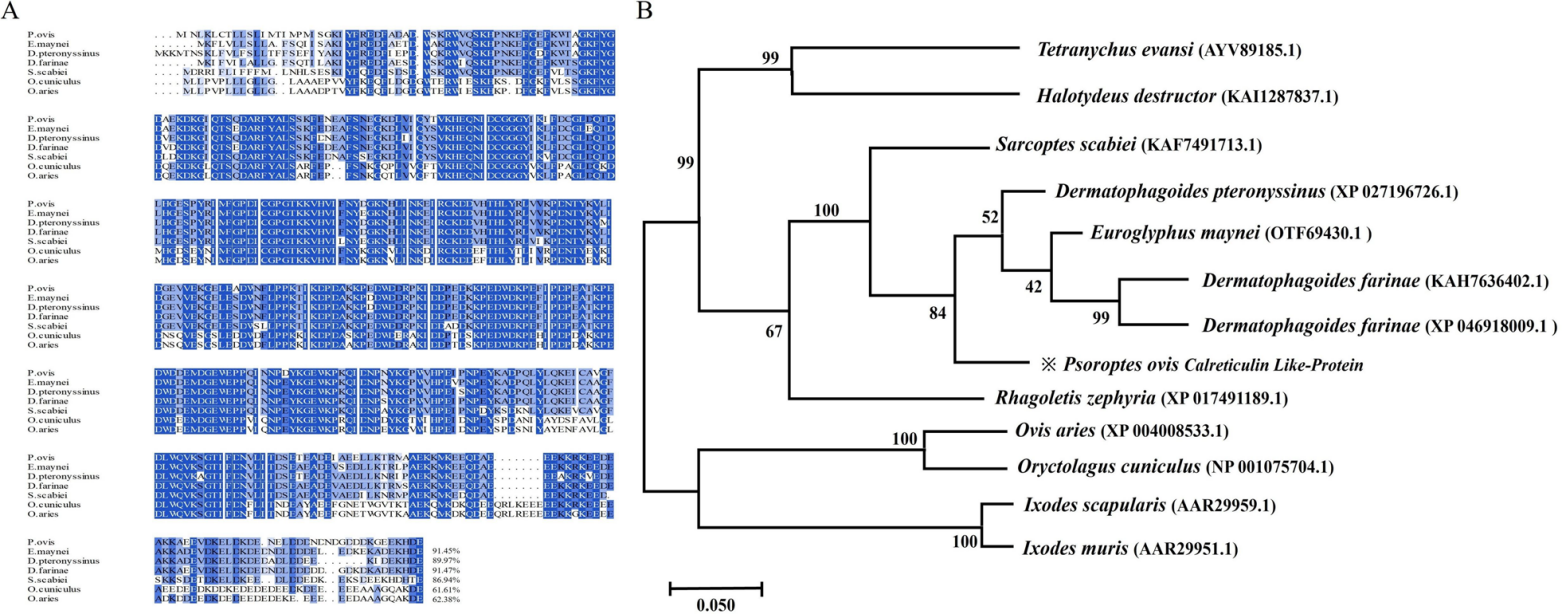
Multiple sequence alignment comparison and phylogenetic analysis of calreticulin amino acid sequences from diverse species. Construction of multiple sequence comparison (**A**) and neighbor-joining (NJ) tree (**B**) based on CRT amino acid sequences. **A** The deduced CRT amino acid sequences were compared with homologous sequences of other mite and host CRT proteins, including *Euroglyphus maynei* (GenBank: OTF69430.1), *Dermatophagoides pteronyssinus* (GenBank: XP_027196726.1), *Dermatophagoides farinae* (GenBank: XP_046918009.1), *Sarcoptes scabiei* (GenBank: KAF7491713.1), *Oryctolagus cuniculus* (rabbit, GenBank: NP_001075704.1) and *Ovis aries* (sheep, GenBank: XP_004008533.1). **B** Construction of NJ tree based on PsoCRT and its homologous amino acid sequence

Prediction of CD4^+^ T-cell epitopes within the PsoCRT amino acid sequence revealed the presence of multiple potential epitopes (Table 2), implying broad immune cell recognition of this protein. Homology analysis, conducted through multiple sequence alignment, revealed a high degree of sequence conservation between PsoCRT and calreticulin sequences of other mite species, including *Euroglyphus maynei*, *Dermatophagoides pteronyssinus*, *Dermatophagoides farinae*, and *Sarcoptes scabiei*, with identity ranges of 86.94–91.45% (Fig. 1A). The neighbor-joining (NJ) tree based on the amino acid alignment of these calreticulins indicated that *P. ovis* has a closer evolutionary relationship to *E. maynei*, *D. pteronyssinus*, and *D. farinae* than to its hosts (sheep and rabbit) (Fig. 1B).

**Table 2:** Prediction of CD4^+^ T-cell epitopes on PsoCRT

Gene	Sequence	Sites	Combined scoring	Immunogenicity score
PsoCRT PQ498351	MTIMPMISGKIYFRE	1–15	43.39092	87.4773
	DARFYALSSKFENEA	61–75	49.22092	88.5523
	KDLVIQYTVKHEQNI	81–95	43.4376	69.594
	GESPYRIMFGPDICG	116–130	43.6444	79.111
	VHVIFNYDGKNHLIN	136–150	37.60064	64.0016
	THLYRLVVKPDNTYK	161–175	48.38148	77.4537

### Western blotting analysis

**Fig. 2:**
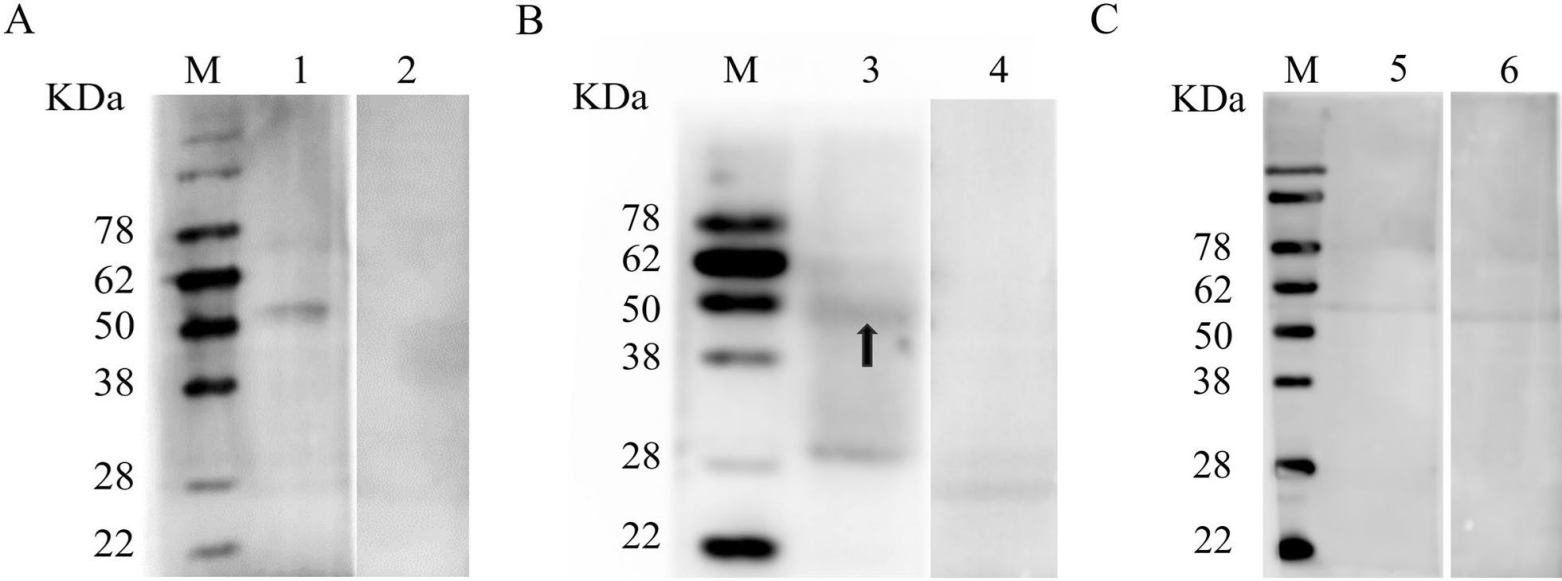
Western blotting analysis of rPsoCRT. **A** Detection of PsoCRT in whole-body protein extracts of *P. ovis* using rat anti-rPsoCRT-IgG serum (lane 1), with a control lane showing negative serum from pre-immunized rats (lane 2). **B** Demonstration of PsoCRT in the E/S proteins of *P. ovis* using the same antibody (lane 3), alongside a corresponding control lane with serum from healthy rats (lane 4). **C** Evaluation of the serological recognition of rPsoCRT by serum from rabbits naturally infested with *P. ovis* (lane 5), compared with a control lane with serum from healthy rabbits (lane 6). Molecular weight standards (lane M) are included in each panel for size reference

Western blotting analysis revealed the presence of PsoCRT native protein in both whole-body proteins and the E/S proteins of *P. ovis* (Fig. 2A and B). This finding indicates that CRT functions as both a structural and a secretory protein in *P. ovis*. Surprisingly, PsoCRT was not recognized by the sera from rabbits infested with *P. ovis* (Fig. 2C).

### Differential transcription levels of PsoCRT mRNA across developmental stages of *P. ovis*

**Fig. 3:**
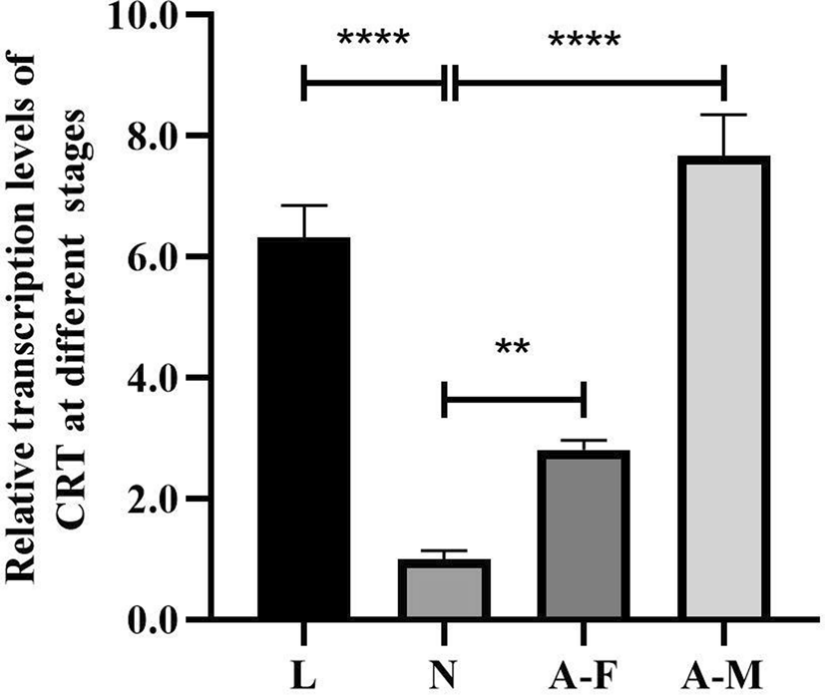
Relative differential expression of PsoCRT genes across various developmental stages of *P.ovis*. Relative PsoCRT gene expression in larvae (L), nymphs (N), adult females (A–F), and adult males (A–M) of *P. ovis* is shown, normalized to β-actin. Data are expressed as mean ± SD. Statistical significance is indicated as follows: **P < 0.01, ****P < 0.0001

qRT-PCR results revealed that PsoCRT mRNA is transcribed throughout the entire lifecycle of *P. ovis*. Notably, the highest transcription levels were observed in male mites, closely followed by larval mites, and then by adult females. The lowest transcript abundance for PsoCRT was detected in the nymph stage, which was approximately sevenfold lower compared with that in adult male mites (*P* < 0.0001) (Fig. 3).

### Native PsoCRT localization in specific epidermal layers of skin lesion in rabbits infested with *P. ovis*

**Fig. 4:**
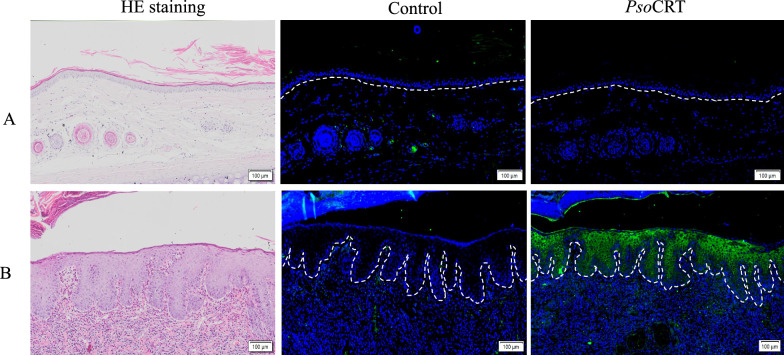
Immunolocalization of native PsoCRT in healthy (**A**) and *P. ovis*-infested (**B**) rabbit ear skin. Left column (HE staining): skin sections were stained with HE for structural analysis. Middle Column (control IgG): sections were incubated with pre-immunization rat IgG as negative control. Right column (anti-rPsoCRT-IgG): sections were incubated with anti-rPsoCRT-IgG for specific detection of native PsoCRT. Scale bar = 100 μm

In the ear skin lesions of rabbits infested with *P. ovis*, native PsoCRT was predominantly localized to the stratum spinosum and stratum granulosum of the epidermis, with no detectable green fluorescence in healthy skin of rabbits. Control immunohistochemical staining using pre-immune rat serum IgG yielded no fluorescent signals in either the lesional or healthy skin tissues (Fig. 4).

### rPsoCRT impacted HaCaT cell proliferation at high-concentration and cell migration at low-concentration

**Fig. 5:**
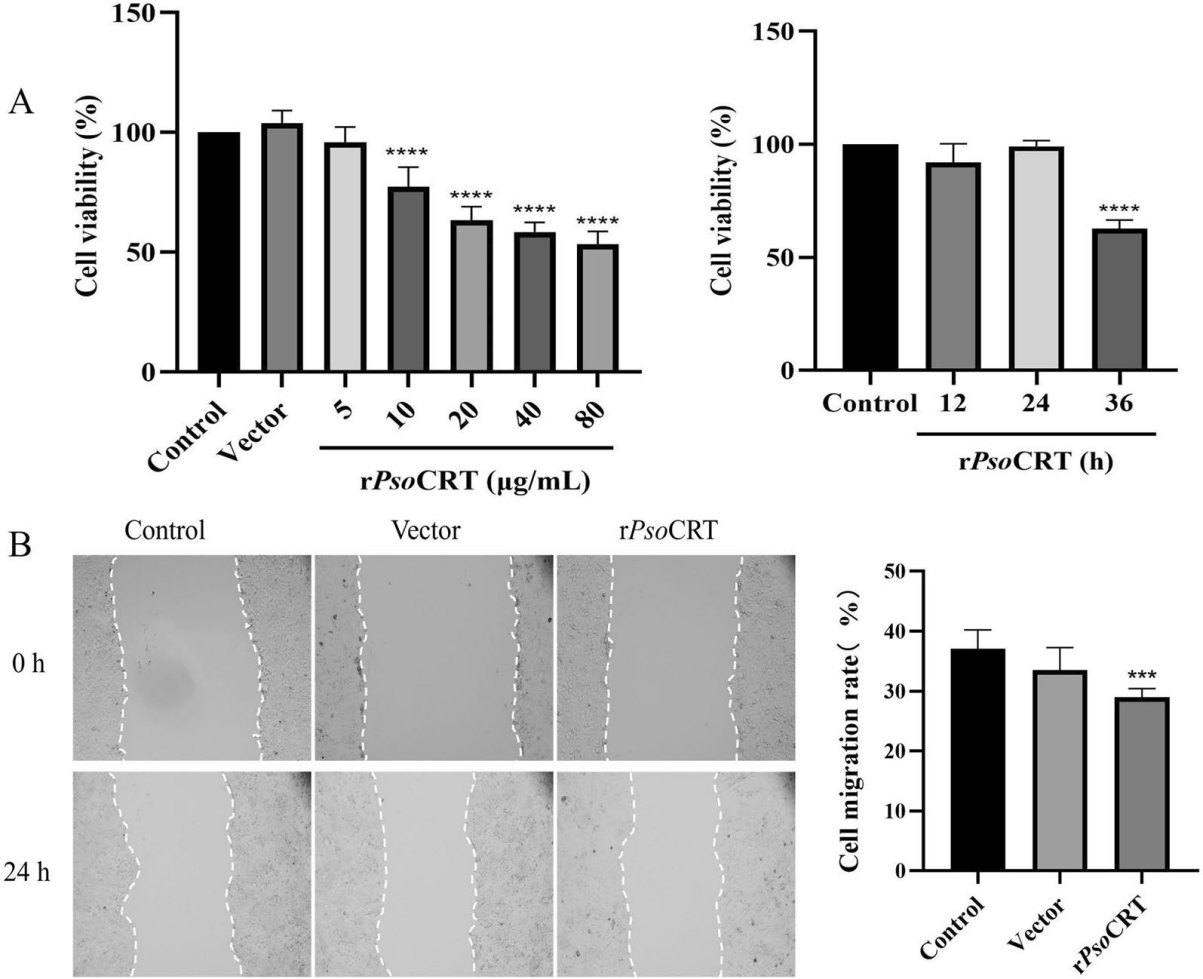
Effects of rPsoCRT on the proliferation and migration of HaCaT cells. **A** Cell proliferation viability was assessed using the CCK-8 assay. Left panel: effects of various concentrations of rPsoCRT on HaCaT cells after 24 h. Right panel: impact of 5 μg/mL rPsoCRT on HaCaT cell proliferation viability over different time periods (12, 24, and 36 h). **B** Cell migration was evaluated using a scratch assay. Representative images were captured using an inverted microscope. Data are presented as mean ± SD. Statistical significance is indicated as follows: ***P < 0.001, ****P < 0.0001; no annotation represents no significant difference

Using a CCK-8 assay, we found that rPsoCRT concentrations above 10 μg/mL significantly inhibited HaCaT cell proliferation in a dose-dependent manner at 24 h (Fig. 5A, *P* < 0.0001), while 5 μg/mL had no significant effect (Fig. 5A, *P* > 0.05). Time-course analysis revealed that 5 μg/mL rPsoCRT inhibited proliferation at 36 h (*P* < 0.0001), but not at 12 or 24 h (*P* > 0.05) (Fig. 5A). Thus, for subsequent experiments, we used 5 μg/mL rPsoCRT for 24 h to avoid proliferation interference. In a scratch assay, 5 μg/mL rPsoCRT significantly inhibited HaCaT cell migration (*P* < 0.001), with no significant difference observed between the PBS group and the pET-32a vector group (*P* > 0.05, Fig. 5B).

### rPsoCRT affected immunoregulatory and differentiation gene expression in the HaCaT cells

**Fig. 6:**
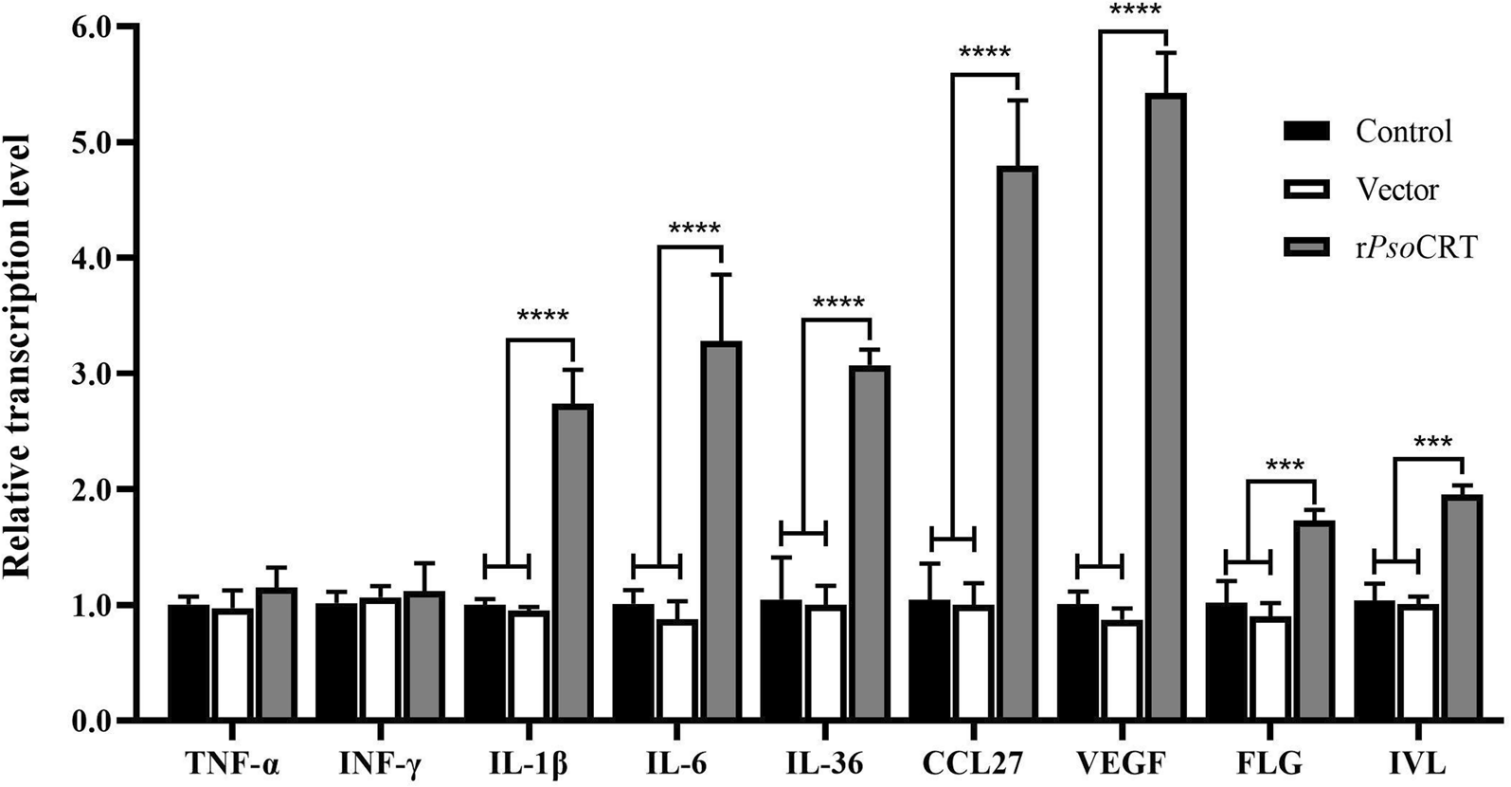
Effect of rPsoCRT on the transcription levels of immune-regulatory genes and differentiation markers in HaCaT cells. Cells were stimulated with 5 μg/mL rPsoCRT for 24 h prior to collection, and the changes in transcription levels of the aforementioned genes were detected using qRT-PCR. Data are presented as mean ± SD. Statistical significance is indicated as follows: ***P < 0.001; ****P < 0.0001; no annotation represents no significant difference

qRT-PCR analysis was conducted to assess the effect of rPsoCRT on the transcriptional profiles of immunoregulatory factors and differentiation marker genes in HaCaT cells. Our results revealed no significant alterations in the expression of all target genes between the PBS group and the pET-32a group (*P* > 0.05) (Fig. 6). Notably, rPsoCRT treatment led to a significant upregulation of IL-1β, IL-6, IL-36, CCL27, and VEGF (*P* < 0.0001) transcripts in HaCaT cells compared with both the PBS and pET-32a groups. Among these, CCL27 and VEGF exhibited the most pronounced increase, with transcript levels approximately fivefold higher than those in the controls, followed by IL-6, which was elevated by approximately threefold. Conversely, TNF-α and IFN-γ transcript levels did not significantly differ across the PBS, pET-32a, and rPsoCRT-treated groups (*P* > 0.05) (Fig. 6). In addition, rPsoCRT treatment resulted in a significant increase in the mRNA levels of the differentiation markers FLG and IVL in HaCaT cells (*P* < 0.001) (Fig. 6).

### rPsoCRT induced histological changes including hyperkeratosis and immune cell infiltration in rabbit ear epidermis

**Fig. 7:**
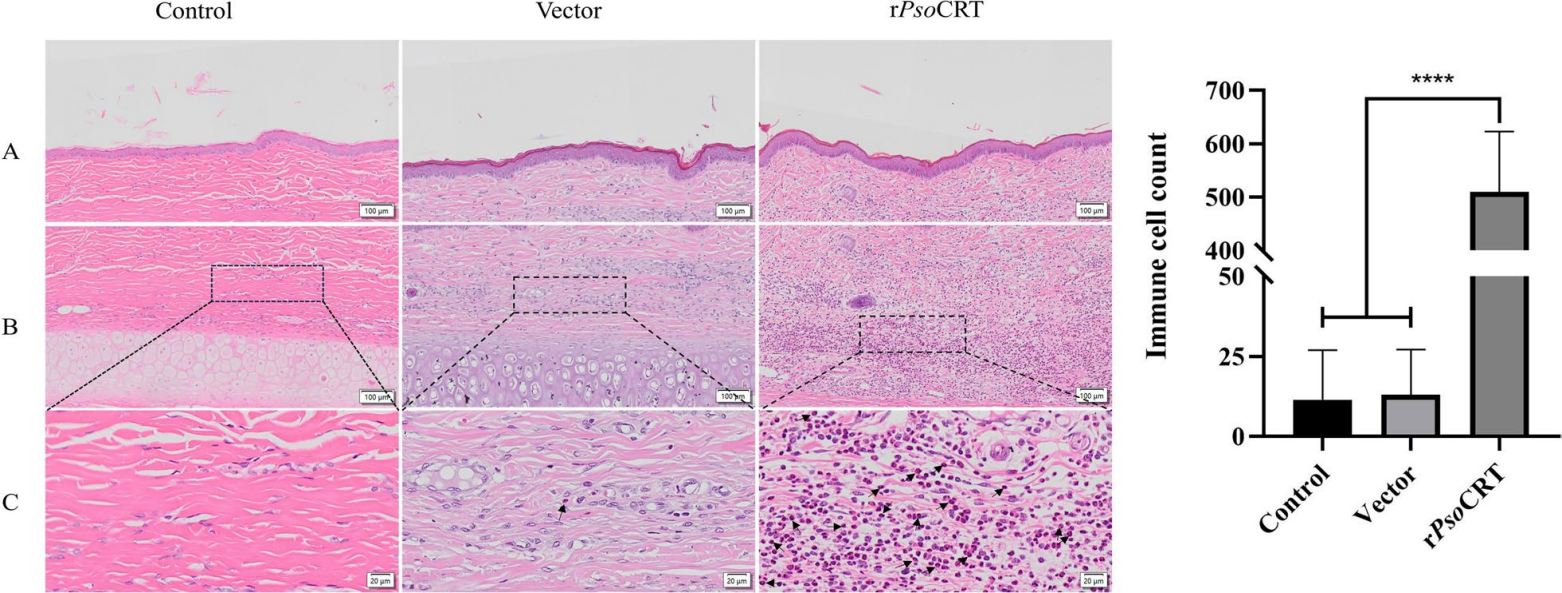
Histopathological observation and immune cell infiltration in rabbit ear skin (HE staining). **A** superficial skin layer; **B** deep skin layer; **C** localized deep skin tissue magnification. Scale bar = 100 μm/scale bar = 20 μm. Data are expressed as mean ± SD. Statistical significance is indicated as follows: ****P < 0.0001, no annotation represents no significant difference. Black arrows: immune cells

After 24 h of intradermal injection of rPsoCRT into rabbit ears, skin tissue sections from the injection site were stained with HE for observation. No significant differences were observed between the PBS and pET-32a groups (*P* > 0.05, Fig. 7). However, in contrast to both control groups, the epidermal tissue of rabbit ears in the rPsoCRT-treated group exhibited hyperkeratosis, along with significant immune cell infiltration in the deeper layers of the skin (*P* < 0.0001, Fig. 7).

### rPsoCRT affected immunoregulatory and differentiation marker gene expression in the rabbit ear skin epidermis

**Fig. 8:**
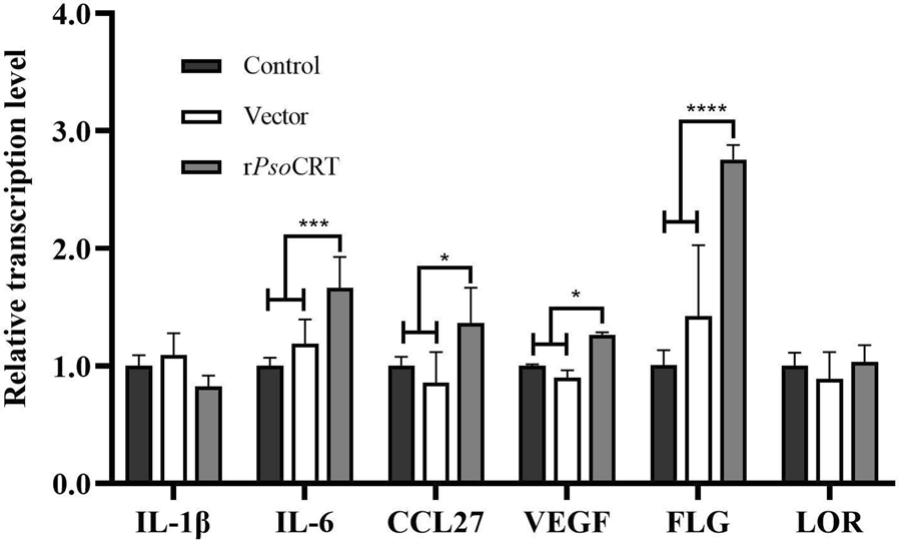
Effect of rPsoCRT on the transcript levels of immunizing factors and differentiation proteins in the healthy rabbit epidermal layer. Data are expressed as mean ± SD. *P < 0.05, ***P < 0.001, ****P < 0.0001, and no annotation represents no significant difference

Our in vitro cellular experiments have previously demonstrated that rPsoCRT upregulates the transcription of immunomodulatory factors and differentiation markers in keratinocytes. To further extend these observations, we conducted an investigation to determine whether rPsoCRT similarly influences the transcription levels of these factors within the epidermal layer of rabbit ear skin. Using qPCR, we found that the administration of rPsoCRT led to statistically significant increases in the transcription levels of IL-6 (*P* < 0.001), CCL27 (*P* < 0.05), VEGF (*P* < 0.05), and FLG (*P* < 0.0001) at the injection sites compared with the control groups. Conversely, no significant alterations were observed in the transcription levels of IL-1β and LOR (Fig. 8).

## Discussion

Calreticulin (CRT) is a multifunctional, conserved protein with a complex structure and diverse functional domains [13]. It plays roles in antigen presentation, immune regulation, and other functions in parasites [22–24]. However, the functional characteristics of CRT protein in *P. ovis* remain unexplored. Here, we functionally characterized a novel CRT in *P. ovis*, termed PsoCRT, and investigated its regulatory role in keratinocytes. We found that PsoCRT, secreted by *P. ovis* into the lesion area, modulated keratinocyte functions to disrupt the skin’s physical and immune barriers, thereby promoting psoroptic mange development.

Studies have demonstrated that CRT is expressed in *Entamoeba histolytica* and *Caenorhabditis elegans* [25, 26], with evidence indicating that the absence of CRT results in impaired growth and development in these organisms [26]. Similarly, *P. ovis* expresses CRT throughout its entire life cycle (Fig. 3), suggesting a potential role for CRT in the growth and development of this parasite, as seen in other organisms. In *C. elegans*, the lack of CRT has been shown to result in decreased mating behavior and defective sperm development, thereby hindering male growth and reproduction [26]. Notably, our observations revealed that CRT transcripts are most abundant in males *P. ovis*, hinting at a possible association with male reproduction and development in this species as well. In addition, we found that PsoCRT was excreted and secreted by *P. ovis* directly into the skin lesion areas on the host’s body surface. Strikingly, rPsoCRT showed no detectable antibody reactivity in sera from *P. ovis-*infested rabbits (Fig. 2), a result consistently replicated in three independent experiments. This immunological feature aligns with documented properties of CRT in *Sarcoptes scabiei*, which evaded antibody recognition in infested host sera [27]. A previous study has hypothesized that *S. scabiei* CRT protein may function as a non-antigenic molecule, with its inability to elicit antibody response potentially attributable to epitope masking through post-translational modifications. This adaptation collectively represent an immune evasion strategy that enables the parasite to circumvent host immune defenses. Specifically, PsoCRT’s failure to induce humoral immunity—through epitope concealment—prevents its detection by the host’s adaptive immune response, thereby facilitating immune evasion.

Keratinocytes are crucial in maintaining skin homeostasis. They meticulously regulate their proliferation, differentiation, and migration within the epidermis, thereby sustaining the integrity and regenerative potential of the skin barrier, as well as maintaining the dynamic balance of the epidermal architecture [28, 29]. However, exposure to exogenous adverse factors or stimulation by external substances can disrupt this delicate balance, leading to functional abnormalities, impaired skin barrier function, and ultimately triggering various skin diseases such as psoriasis, eczema, and atopic dermatitis [30, 31]. In our present study, we have uncovered that during parasitism, *P. ovis* secretes and excretes native CRT protein into the epidermal spinous and granular layers of skin lesions (Fig. 4), which are primarily composed of keratinocytes. This suggests that *P. ovis* can directly target keratinocytes with its CRT protein during its life cycle. We hypothesize that CRT, upon interacting with keratinocytes, may modulate their function. Indeed, using an in vitro model with HaCaT cells, we found that rPsoCRT significantly inhibited the proliferation and migration of keratinocytes (Fig. 5), implying that *P. ovis* might inhibit these process in the host epidermis by secreting PsoCRT, thereby hindering skin wound healing and ultimately leading to defects in the host skin’s physical barrier. To validate this hypothesis, we conducted in vivo experiments conducted on New Zealand White rabbits and found that injection of rPsoCRT resulted in abnormal expression of FLG, a differentiation protein crucial for the skin’s physical barrier (Fig. 8), further causing dysfunction of the physical barrier properties of the rabbits’ skin. A defective skin physical barrier facilitates the infiltration of mite allergen molecules, exacerbating the inflammatory response [32, 33]. This, in turn, leads to increased inflammatory exudate production, providing more food sources for the mites [34, 35], resulting in an increase in mite populations and further progression of the psoroptic mange. In addition, our study revealed that rPsoCRT significantly promoted the transcriptional expression of key differentiation-related markers in keratinocytes, with notable upregulation of *IVL* and *FLG* (Fig. 6). In vitro experiments also demonstrated that rPsoCRT resulted in significantly elevated expression of FLG in the epidermis of rabbits, furtherly leading to hyperkeratinization at the injection sites of rabbits. Notably, severe hyperkeratosis was observed in the cases of natural *Psoroptes* infestation [36]. These findings indicate that CRT secreted by *P. ovis* contributes to the pathogenesis of psoroptic mange.

Keratinocytes, as integral components of the skin’s immune barrier, secrete immune-regulatory factors that collaborate with immune cells and bioactive molecules to defend against pathogens and maintain skin health [37]. In the context of parasitic infestations, CRT of parasites has been implicated in modulating the host immune system and is involved in the parasitic disease initiation and progression, including the establishment of parasite infestation, the immune evasion strategies employed by parasites, and their ability to infiltrate tissue [16, 17, 38]. In *P. ovis*, we revealed that rPsoCRT significantly enhances the transcription of *IL-1β*, *IL-6*, *IL-36*, *CCL27*, and *VEGF* in keratinocytes (Fig. 6). This upregulation facilitates the recruitment and infiltration of immune cells to the infection site [39, 40]. The elevated expression of *CCL27*, which binds to CC chemokine receptor 10 (CCR10), might promote the migration of immune cells to specific skin regions [41], while increased VEGF expression might enhance vascular permeability [42], enabling immune cells extravasation from blood vessels into localized skin areas. This process, in turn, disrupts the delicate balance of local immune homeostasis [43]. In vivo experiments corroborate these hypotheses, with rabbit ear skin injected with rPsoCRT exhibiting high expression of *IL-6*, *CCL27*, and *VEGF* in the epidermis, accompanied by extensive immune cell infiltration in the dermis (Figs. 7, 8). These findings demonstrate that PsoCRT might employ a dual mechanism to disrupt epidermal homeostasis, representing an evolved parasitic strategy that both suppress keratinocyte proliferation/migration and induces inflammatory responses in HaCaT cells, thereby simultaneously compromising both physical and immune barriers. This coordinated targeting of distinct host defense systems creates a permissive microenvironment for *P. ovis* colonization, where impaired re-epithelialization prevents wound closure concurrent with excessive inflammation generating nutrient-rich exudates that sustain mite populations—collectively facilitating parasite establishment and driving chronic psoroptic mange progression.

## Conclusions

PsoCRT, secreted by *P. ovis*, disrupted keratinocyte physical and immune barriers, resulting in impaired skin dysfunction and creating a conducive microenvironment for *P. ovis* parasitization, thereby playing an important role in psoroptic mange pathogenesis*.*

## Data Availability

No datasets were generated or analyzed during the current study.

## Abbreviations

*P. ovis*: *Psoroptes ovis*
PsoCRT: Calreticulin of *P. ovis*
rPsoCRT: Recombinant PsoCRT
DMEM: Dulbecco’s modified Eagle medium
FBS: Fetal bovine serum
LB: Luria–Bertani medium
NJ: Neighbor-joining
E/S proteins: Excretory–secretory proteins
IPTG: Isopropyl-β-D-thiogalactoside
PCR: Polymerase chain reaction
PBS: Phosphate-buffered saline solution
SDS-PAGE: Sodium dodecyl sulfate–polyacrylamide gel electrophoresis
HRP: Horseradish peroxidase
TBST: Tween 20 in Tris-buffered saline
qRT-PCR: Real-time fluorescence quantitative PCR
HE: Hematoxylin and eosin staining
FITC: Fluorescein isothiocyanate
DAPI: 4′, 6-diamidino-2-phenylindole
CCK-8: Cell Counting Kit-8 assay
HaCaT: Human keratinocyte cells
IVL: Involucrin
FLG: Filaggrin
TNF-α: Tumor necrosis factor-α
IFN-γ: Interferon-γ
IL-1β: Interleukin-1 beta
IL-6: Interleukin-6
IL-36: Interleukin-36
CCL27: C–C motif chemokine ligand 27
VEGF: Vascular endothelial growth factor
GAPDH: Glyceraldehyde-3-phosphate dehydrogenase
SD: Standard deviation
LOR: Loricrin
CCR10: CC chemokine receptor 10
